# Improving adaptive response to negative stimuli through non-emotional working memory training

**DOI:** 10.3389/fnbeh.2022.1058866

**Published:** 2023-01-05

**Authors:** Quanshan Long, Linlin Yu, Yancheng Tang, Qing Li, Na Hu, Yan Gu, Antao Chen

**Affiliations:** ^1^Faculty of Education, Yunnan Normal University, Kunming, China; ^2^Key Laboratory of Cognition and Personality of Ministry of Education, Faculty of Psychology, Southwest University, Chongqing, China; ^3^Department of Preschool & Special Education, Kunming University, Kunming, China; ^4^School of Psychology, Shanghai University of Sport, Shanghai, China

**Keywords:** working memory, emotional response, cognitive training, expectation, placebo effect

## Abstract

People with high working memory (WM) capacity tend to respond proactively and experience a decrease in undesired emotions, implying the potential influence of WM training on emotional responses. Although training emotional WM could enhance emotional control, the training also improves emotional response itself. Thus, the far-transfer effects of non-emotional WM training on emotional responses remain an open question. In the present study, two experiments were conducted to detect these effects. The Preliminary experiment matched the expectations of the gains of the training tasks between the experimental and active control groups (*n* = 33). In Experiments 1 and 2, participants performed 7-day and 15-day training procedures, respectively. Results indicated that after a 7-day training, non-emotional WM training (*n* = 17) marginally reduced individuals’ emotional responses compared with the active control group (*n* = 18); importantly, this improvement became significant after a 15-day training (*n*_(WM training)_ = 20, *n*_(active control)_ = 18). A combination analysis for Experiments 1 and 2 showed that training gains on WM performance were significantly related to reduced emotional responses (*r* = −0.359), indicating a dosage effect. Therefore, non-emotional WM training provides a safe and effective way to enhance adaptive emotional responses.

## Introduction

Negative emotional responses can be activated by internal or external negative stimuli (Carretie et al., 2001; Ochsner et al., 2009). Individuals have a tendency to respond more intensely to negative information than positive information in daily life (Ito and Cacioppo, 2005; Long et al., 2015). Excessive negative emotional response is not conducive to individuals’ physical and mental health, and researchers try to find specific tactics to increase adaptive responses to negative stimuli.

It is well known that increasing adaptive responses rely on a balance of top-down cognitive control processes with bottom-up emotion-related responses (Ochsner and Gross, 2005). As indicated by previous studies, high working memory (WM) could predict the emotional response (Schmeichel and Demaree, 2010; Coifman et al., 2021; Kobayashi et al., 2021; Garrison and Schmeichel, 2022) and was associated with optimal adaptation in emotion perception (Lynn et al., 2016). This implied that enhancing cognitive performance (i.e., cognitive training) may increase the adaptive response to negative stimuli. An inspiring intervention study reinforces this possibility, indicating that after training participants to monitor and update the affective contents of working memory, they learned to suppress emotional responses in the presence of adverse stimuli (Schweizer et al., 2013). However, prior WM training employed negative stimuli, likely to train WM and emotional response. Moreover, repeatedly experiencing negative emotions may be detrimental to human wellness, especially for affective disorders or children (Disner et al., 2011). Negative emotion is difficult to form emotional habituation and even increase undesirable experience (Long et al., 2015). Obviously, training cognition without emotional information would be a safer method to popularize for use with various groups.

Some researchers indicated that cognitive training without emotional information also could reduce emotion-related reactivity (Beauchamp et al., 2016; Cohen et al., 2016). However, cognitive training has sparked heated discussions about its effectiveness (Shipstead et al., 2012; Au et al., 2016). Some studies did not find the transfer effects (Shipstead et al., 2012; De Simoni and von Bastian, 2018), i.e., cognitive training had no significant effect on psychopathology measures (Mewton et al., 2020). Others believed that the training could facilitate the development of new skills, which might transfer to new situations and domains that shared some properties with the training task (Diamond and Lee, 2011; Gathercole et al., 2019). For example, WM training could decrease emotion-related impulsivity (Xiu et al., 2016; Peckham and Johnson, 2018) and psychopathological symptoms (Koster et al., 2017; Beloe and Derakshan, 2020). These inconsistent results imply that the transfer effects of cognitive training need to be clarified.

Notably, individuals’ expectations of improvement may confuse the transfer effects driven by cognitive training (Boot et al., 2013), as expectation has been shown to have a significant effect on cognition, emotion, and behavior (Foroughi et al., 2016; Long et al., 2020). This means that the inconsistent results may come from the low-quality control of expectation, which may induce the placebo effect and cause changes in experimental results. Previous studies use the active control group to rule out the placebo effect (Dougherty et al., 2016). Compared with the passive control group that only receives the pretest and posttest (Jaeggi et al., 2008), setting the active control group is a better way to control undesirable effects because both training and control groups receive the same amount of training. However, the influence of expectations has not been seriously considered in previous studies exploring the gains of cognitive training on emotional response. It is necessary to strictly control expectations to ensure the purity of the transfer effects.

### The current study

In the present study, we conducted two experiments to examine the influence of non-emotional WM training on emotional responses when the expectations between the training and active control groups were matched carefully. A typical dual *n*-back task without emotional information served as the WM training task (Jaeggi et al., 2008). The difficulty was adapted according to participant performance to promote the transfer of training (Flegal et al., 2019). Participants needed to decide whether the current item was identical to the one presented *n* items back in the sequence. Besides, an expectation-matched active control group was included to control placebo effects strictly. The visual search task that exerted minimal demand on the WM served as the training task of the active control group.

Before the formal experiment, we conducted a Preliminary experiment (see Supplementary Material for detail) to match participants’ expectations for improvement between the two groups. In order to find an efficient training method, we determined the training duration based on previous studies. Participants usually perform 8, 10, 14, and 20 training sessions in previous cognitive training studies (Jaeggi et al., 2008; Beauchamp et al., 2016; Hoorelbeke et al., 2016), we first conducted a 7-day (seven sessions) training procedure in Experiment 1. Meanwhile, developing new routines may need long-term training (Gathercole et al., 2019). The training duration was furtherly extended to 15 days in Experiment 2. Finally, a combination analysis for these two experiments allowed us to examine the dosage effect of non-emotional WM training on emotional responses.

Picture stimuli elicited fewer physiological and emotional arousal responses than video stimuli (Courtney et al., 2010). Considering that low-arousal stimuli are more frequently encountered in daily life, we selected emotional pictures from the Chinese Affective Picture System (CAPS; Bai et al., 2005) as the stimuli for the emotion regulation (ER) task. This task included three conditions (Neutral, Attend, Regulate). The *emotional response* is related to the individual’s response to adverse stimuli, represented by the difference between the Attend and Neutral conditions. *Emotion regulation* was represented by the difference between the Regulate and Attend conditions. It is worth noting that there are many emotion regulation strategies in the process model of emotion regulation (Gross, 2015). Given that the reappraisal strategy is more adaptive than other strategies (Gross, 1998), participants in the Regulate condition needed to use it to down-regulate their negative emotions. In this study, a *cue* indicated which of three conditions would be presented. This study examined the transfer effects of non-emotional WM training on emotional response and emotion regulation. The gains of non-emotional WM training would improve individuals’ adaptive response to negative stimuli.

## Experiment 1 A Randomized Placebo-Controlled Trial of 7-Day WM Training

According to the Preliminary experiment in Supplementary Material, the expectations of gains from the WM and visual search task were similar. Thus, the expectations-matched training task was used in Experiment 1, in which the WM training and active control groups had similar expectations. Experiment 1 aimed to examine the effects of non-emotional WM training on the emotional response after strictly controlling expectations.

### Methods

#### Participants

The sample size of Experiment 1 was determined by *a priori* statistical power analyses using G^*^power software (Faul et al., 2007). The power analysis (power ≥ 0.8) on within-between interaction design, assuming a small-to-medium effect size of 0.25, indicated a sample size of 12 for each group. Considering the sample size of previous training studies (16 or fewer), we recruited two independent samples with *n* = 20 each in Experiment 1.

Accordingly, 40 participants were randomly and evenly assigned to the active control group (AC1) and the 7-day WM training group (7-WMT). For the final analysis, we cleaned data based on two criteria: (1) participants discovered the true purpose of training, which might induce expectations of improvement, potentially contaminating the observed results (Long et al., 2020). In order to achieve this criterion, participants needed to finish a questionary after the posttest in which they were asked to describe the purpose of the study they thought; and (2) participants did not complete the training requirements. Their data were discarded to ensure all participants completed the same length of training sessions. Therefore, two AC1 participants were excluded because they did not complete the entire seven days of training. In the 7-WMT group, one participant was excluded for failing to complete the whole training, and two were excluded for discovering the purpose of the training. Thus, 35 participants (10 men, 20.23 ± 1.52 years) were submitted to the final statistical analysis. There was no significant difference between the 7-WMT and AC1 groups regarding sex distribution, age, Beck Depression Inventory, and Spielberger State-Trait Anxiety Scale scores (*p*_s_ > 0.05).

#### Stimuli and design

**Figure 1:**
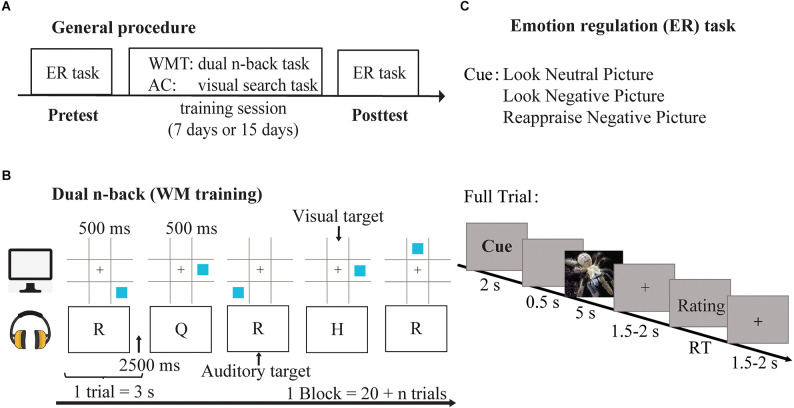
General procedure **(A)** and experimental tasks for Experiments 1 and 2. Task design for WM training, illustrated by a dual *n*-back condition, presented the visual stream at the same rate as the auditory stream **(B)**. Task design of emotion regulation **(C)**.

The 7-WMT and AC1 groups performed all tasks in the lab with similar training settings (Figure 1A). After the pretest, participants completed a 7-day training intervention of approximately 25 min each day. The posttest assessment and questionnaire were performed on the first or second day after the completed training. Each participant received a payment of 180 RMB (approximately 26 US dollars) at the end of the study.

Participants were told that this was a comprehensive study and measured their brain responses to a specific task. None of the participants received explicit or implicit information regarding the actual purpose of the training from recruitment to the end of the study. At the end of the experiment, we assessed participants’ expectations and motivation *via* a questionnaire.

(1) **Adaptive dual *n*-back training task**. The 7-WMT group was trained on a computerized dual *n*-back task (Figure 1B)^1^. In this task, a sequence of squares was presented at eight different locations on a computer screen at a mean rate of 3,000 ms (stimulus length, 500 ms; inter-stimulus time, 2,500 ms). Simultaneously, one of eight consonants was presented with each square. Participants were required to press a key (“A” for the position match; “L” for the audio match) when either the position or word from the current stimulus matched the stimulus presented *n* items back. Each block included six auditory and six visual targets.
  We conducted an adaptive training approach in which the level of *n* (e.g., *n* = 2, 3, 4) would vary as a function of the participant’s performance in the previous block. The participants started training at a 2-back level to promote learning experimental rules. If the rate of correct answers was 80% or above, the level of *n* was increased in the next block. The level was decreased if the scores of the three blocks were equal to 50% or below. In other conditions (51% to 79%), *n* was maintained. One training session comprised 20 blocks (approximately 25 min), and each block consisted of 20 + *n* trials. Participants began at the same *n*-back level as they had left off in the next training session.
(2) **Active control task**. The AC1 group trained on a visual search task (Anderson et al., 2011), and the training session was similar to the 7-WMT group. A fixation display was presented for a randomly varying interval (400, 500, or 600 ms), followed by a search display that remained on the screen until a decision was made or the display timed out (1,500 ms). The participants pressed a key when identifying the horizontally (F) or vertically (J) orientated target in the diamond among circles. The participants completed three blocks during each training session, totaling 216 trials (approximately 20 min).
(3) **Emotion regulation (ER) Task**. This experimental procedure was similar to a prior study (Long et al., 2020), in which 96 negative emotional pictures and 48 neutral pictures were selected from the CAPS. The negative emotional pictures included some adverse contents like snakes, wars, and car accidents. The mean valence of negative emotional pictures was 2.17; the mean arousal was 5.83. The neutral picture involved neutral emotional contents like tables and chairs. Furthermore, the mean valence of the neutral picture was 5.11; the mean arousal was 4.25. All pictures were randomly assigned to pretest and posttest sets. The two sets were similar in valence and arousal (*p*_s_ > 0.05).

Each ER task involved three blocks of 36 trials each, totaling 108 trials. Participants needed 20 min to finish the pretest or posttest. One of three *cues* was presented before each trial, resulting in three conditions: Neutral, Attend, and Regulate. Specifically: (a) *Look Neutral Picture* (Neutral), participants attended to non-emotional stimuli, which would not induce a specific emotional response; (b) *Look Negative Picture* (Attend), participants passively attended to the emotional stimuli so their natural emotional response to them could be evoked and measured; and (c) *Reappraise Negative Picture* (Regulate), participants viewed emotionally negative pictures and downregulated their emotions using a reappraisal strategy (Yuan et al., 2015). In practice, participants needed to understand the cue-task association and corresponding demands.

Three types of experimental trials were randomly presented in the ER task. A cue of 2,000 ms initiated each *Full Trial* (Figure 1C). An interval was presented for 500 ms, followed by a 5,000 ms picture. Next, a blank screen was presented for a random duration between 1,500 and 2,000 ms. Participants then rated their negative emotions on a 5-point scale (1 = *not at all negative*; 5 = *extremely negative*). Finally, a 1,500–2,000 ms inter-trial interval was presented. The *Anticipation Only trial* involved the cue, anticipatory interval (1,500–2,000 ms), and rating display. The *Stimulus Only Trial* was similar to the Full Trial, while an emotional picture followed the cue. Data analysis was conducted on the averages of the Full Trial and the Stimulus Only Trial to investigate the emotional response to negative stimuli. Crucially, the emotional report of the Anticipation Only Trial could index proactive emotion regulation as the emotional stimuli were absent in these trials.

#### Data analysis

We performed a series of repeated analyses of variance (ANOVA) designed measures to identify the transfer effect of WM training on emotional response. First, the pretest performance was evaluated by a repeated measures ANOVA, with Cue (Neutral, Attend, and Regulate) as a within-subjects variable and Group (7-WMT, AC1) as a between-subjects variable. Training-related changes were then tested *via* the paired-sample *t*-tests in corresponding training tasks (WM task, search task). In order to increase the signal-to-noise ratio, the mean performance of the first two training sessions served as the pre-training performance; the mean performance of the last two training sessions served as the post-training performance (Jaeggi et al., 2011). Finally, two ANOVAs were used to explore the training-related changes in emotional response and emotion regulation, with Test Session (pretest, posttest) and Cue (Neutral, Attend/Regulate) as a within-subjects variable, and Group (7-WMT, AC1) as a between-subjects variable. The degree of freedom was corrected using Greenhouse-Geisser correction whenever the assumption of sphericity was violated. *Post hoc* multiple comparisons were adjusted using Holm-Bonferroni correction.

### Results

#### Pretest performance

Participants rated their extent of compliance with instructions on a seven-point scale (1 = *not at all*; 7 = *fully compliant*) after the pretest. We conducted a one-sample *t*-test on the compliance rating, which indicated that all participants followed the instructions of the Neutral (6.34), Attend (6.34), and Regulate (6.03) conditions (*p*_s_ < 0.001).

**Figure 2:**
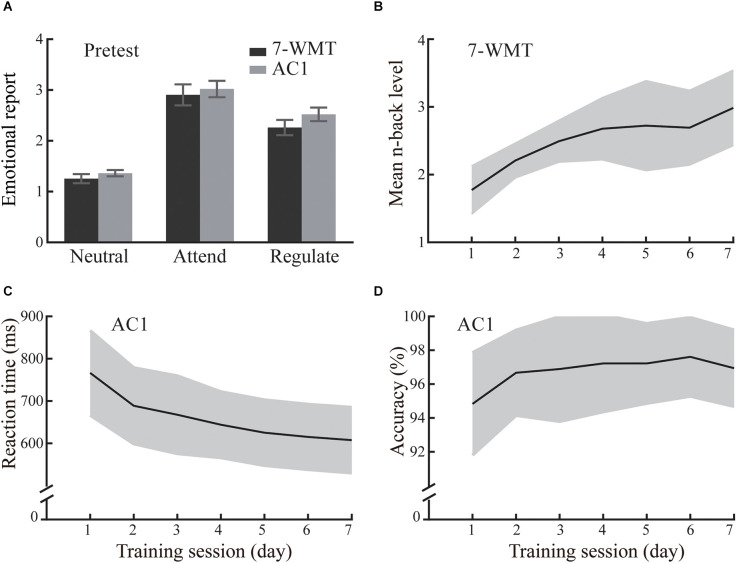
The emotional reports and training performance of the 7-day WM training group (7-WMT) and active control group (AC1) in Experiment 1. The 7-WMT and the AC1 group experienced similar emotions at the pretest **(A)**. Error bars represent standard errors. The improved performance in the trained task is shown separately for the 7-WMT group **(B)** and the AC1 group **(C,D)**. The shading around the solid line indicates the standard deviation.

At the pretest (baseline), we found no significant main effect of Group (*F*_(1,33)_ = 0.99, *p* = 0.328) or Group × Cue interaction (*F*_(2,66)_ = 0.35, *p* = 0.708). However, there was a significant main effect of Cue (*F*_(2,66)_ = 137.83, *p* < 0.001, $\eta_{p}^{2}$ = 0.807). The Attend condition (2.96) induced more negative emotional reports than the Neutral condition (1.31, *p* < 0.001). Compared with the Attend condition, participants in the Regulate condition more effectively regulated their emotional reports using the reappraisal strategy (2.39, *p* < 0.001). Together, these results indicated that both groups responded similarly to negative emotion at the pretest (Figure 2A).

#### Training related changes after a 7-day training

There were significant improvements in the WM and active control tasks (Figures 2B–D). The 7-WMT group showed a significant increase in the performance of the *n*-back task from pre-training to post-training (1.99 vs. 2.84, *t*_(16)_ = 6.78, *p* < 0.001, *Cohen’s d* = 3.39). Similarly, the AC1 group also showed better performance from pre-training to post-training. They displayed better performance in terms of accuracy (97.3% vs. 95.8%, *t*_(17)_ = 4.47, *p* < 0.001, *Cohen’s d* = 2.17) and response speed (611 ms vs. 728 ms, *t*_(17)_ = 13.87, *p* < 0.001, *Cohen’s d* = 6.76) at post-training compared to pre-training. These results suggested that training could change the performance of the task.

**Figure 3:**
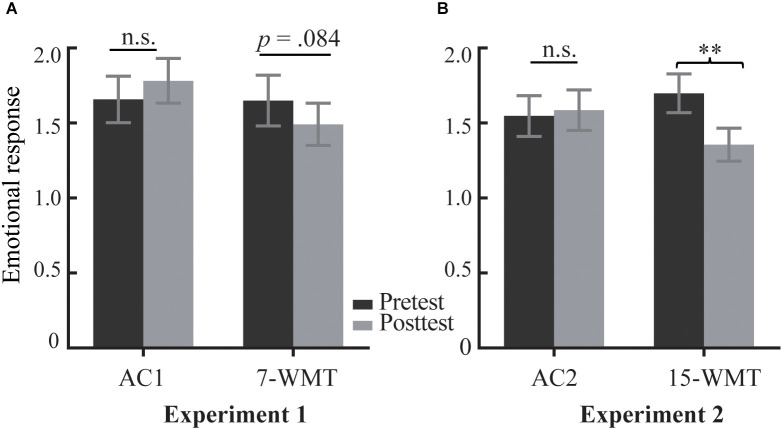
Transfer effects from WM training to emotional response in Experiment 1 and Experiment 2. Means and standard errors of emotional response (Attend − Neutral) for the 7-day/15-day WM training group (7-WMT/15-WMT) and the active control group (AC1/AC2) at the pretest and posttest in Experiments 2 **(A)** and 3 **(B)**. ^**^p < 0.01; n.s., non-significant; WM, working memory.

Table 1 summarizes the emotional reports for the three conditions. First, a three-way ANOVA was performed for the transfer effect on emotional response, demonstrating a significant three-way interaction (*F*_(1,33)_ = 4.63, *p* = 0.039, $\eta_{p}^{2}$ = 0.123). Subsequent analysis displayed a marginally significant pretest to posttest decrease in emotional response (Attend—Neutral) in the 7-WMT group (*F*_(1,16)_ = 3.39, *p* = 0.084; Figure 3A). However, the AC1 group showed no significant improvement from the pretest to posttest assessment (*F*_(1,17)_ = 1.59, *p* = 0.224). Next, we analyzed the transfer effect on emotion regulation and found that the Test Session (pretest, posttest) × Group (7-WMT, AC1) × Cue (Attend, Regulate) interaction was not significant (*F*_(1,33)_ = 1.12, *p* = 0.297).

**Table 1:** The emotional reports for the neutral, attend, and regulate conditions in Experiment 1.

	**Pretest (Mean ± SD)**		**Posttest (Mean ± SD)**	
**Cue**	**7_WMT**	**AC1**	**7_WMT**	**AC1**
Neutral	1.25 ± 0.38	1.36 ± 0.26	1.16 ± 0.18	1.39 ± 0.26
Attend	2.90 ± 0.85	3.02 ± 0.69	2.65 ± 0.69	3.18 ± 0.64
Regulate	2.26 ± 0.62	2.52 ± 0.56	2.09 ± 0.68	2.59 ± 0.59

Note: SD, standard deviations; 7_WMT, 7-day working memory training group; AC1, active control group.

## Experiment 2 A Randomized Placebo-Controlled Trial of 15-Day WM Training

In Experiment 1, we did not find the significant transfer effects of 7-day non-emotional WM training on emotional response. In this experiment, we extended the number of training sessions to 15 days and aimed to test whether training non-emotional WM could significantly improve participants’ adaptive response to negative stimuli. Besides, we attempted to examine the dosage effect of non-emotional WM training on emotional response using a combined analysis for Experiment 1 and Experiment 2.

### Methods

#### Participants

Forty new participants were recruited and randomly assigned to the 15-day WM training group (15-WMT) and the active control group (AC2). Two participants in AC2 group were excluded from the final analysis because they did not meet the training requirements. Ultimately, 38 participants (11 men, 20.26 ± 1.16 years old) were used in the final data analysis. The two groups did not differ in age, sex distribution, or emotion-related states (anxiety and depression; *p*_s_ > 0.05). After completing the experiment, each participant received 260 RMB (approximately 38 US dollars).

#### Stimuli and design

The general procedure and experimental tasks of Experiment 2 were identical to those in Experiment 1 (Figure 1). The lab-based training was increased from 7 to 15 days.

#### Data analysis

Similar to Experiment 1, this experiment used a series of ANOVAs designs to explore the transfer effects of non-emotional WM training on emotional responses and emotional regulation, respectively. Then, a combination analysis for Experiment 1 and Experiment 2 was conducted. Data analysis was performed using the Pearson correlation coefficient and ANOVA.

### Results

#### Pretest performance

The analysis of compliance with instructions showed that the participants wholly followed the instructions for the Neutral (6.17), Attend (6.17), and Regulate conditions (5.92; *p*_s_ < 0.001).

**Figure 4:**
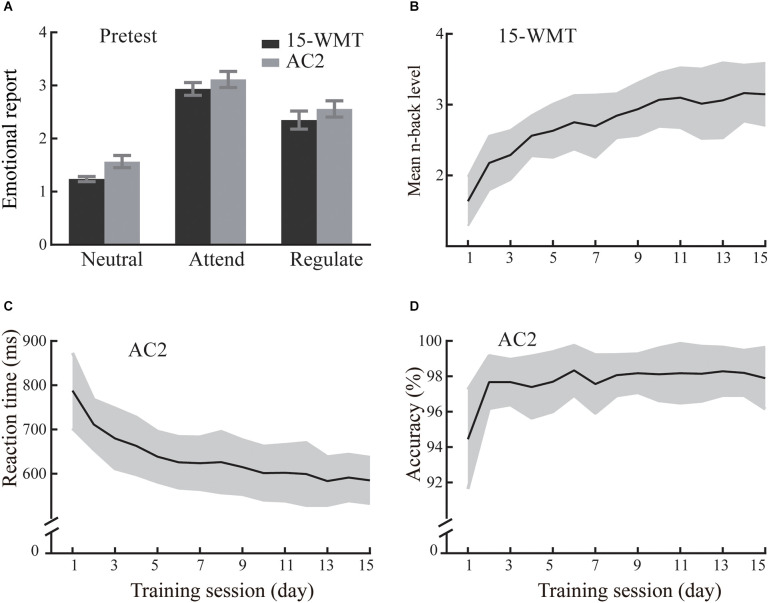
The emotional reports and training performance of the 15-day WM training group (15-WMT) and active control group (AC2) in Experiment 2. The mean emotional reports and error bars for three conditions at the pretest **(A)**. Training-related changes in the 15-WMT **(B)** and AC2 **(C,D)**. Shading indicates standard deviation.

At the pretest, the 15-WMT and AC2 groups reported similar levels of negative emotion (Figure 4A). Specifically, there was no significant main effect of Group (*F*_(1,36)_ = 2.91, *p* = 0.096) or Group (15-WMT, AC2) × Cue (Neutral, Attend, Regulate) interaction (*F*_(2,72)_ = 0.28, *p* = 0.758). The main effect of Cue was significant (*F*_(2,72)_ = 117.95, *p* < 0.001, $\eta_{p}^{2}$ = 0.766). Compared to the Neutral condition (1.40), participants reported more negative emotions in the Attend condition (3.03, *p* < 0.001). Besides, they could use a reappraisal strategy to effectively downregulate their negative emotions in the Regulate condition (2.45) relative to the Attend condition (*p* < 0.001).

#### Training-related changes after a 15-day training

With practice, the 15-WMT and AC2 groups showed continuous improvement in the corresponding training tasks (Figures 4B–D). Consistent with Experiment 1, the 15-WMT group displayed a significant pre-training to post-training increase on the *n*-back task (1.91 vs. 3.15, *t*_(19)_ = 15.35, *p* < 0.001, *Cohen’s d* = 7.04). Moreover, the AC2 group showed significant improvement from pre-training to post-training in accuracy (96.1% vs. 98.0%, *t*_(17)_ = 5.85, *p* < 0.001, *Cohen’s d* = 2.84) and reaction time (750 vs. 588 ms, *t*_(17)_ = 10.12, *p* < 0.001, *Cohen’s d* = 4.91). They displayed higher accuracy and faster response time at the post-training than pre-training.

Table 2 shows the emotional reports for the three experimental cues. Similar to Experiment 1, the results indicated that the interaction of the Test Session (pretest, posttest) × Group (15-WMT, AC2) × Cue (Neutral, Attend) was significant (*F*_(1,36)_ = 6.44, *p* = 0.016, $\eta_{p}^{2}$ = 0.152); the interaction of the Test Session (pretest, posttest) × Group (15_WMT, AC2) × Cue (Attend, Regulate) was not significant (*F*_(1,36)_ = 0.18, *p* = 0.677). As shown in Figure 3B, compared to the pretest, the 15-WMT group showed a significant reduction in emotional response (Attend − Neutral) at the posttest (*F*_(1,19)_ = 10.89, *p* = 0.004, $\eta_{p}^{2}$ = 0.364). Conversely, the AC2 group showed no significant pretest to posttest change in emotional response (1.55 vs. 1.59, *F*_(1,17)_ = 0.13, *p* = 0.726).

**Table 2:** The emotional reports for the Neutral, Attend, and Regulate conditions in Experiment 2.

	**Pretest (Mean ± SD)**		**Posttest (Mean ± SD)**	
**Cue**	**15_WMT**	**AC2**	**15_WMT**	**AC2**
Neutral	1.24 ± 0.21	1.57 ± 0.49	1.20 ± 0.26	1.39 ± 0.28
Attend	2.94 ± 0.54	3.11 ± 0.65	2.55 ± 0.57	2.98 ± 0.73
Regulate	2.35 ± 0.76	2.56 ± 0.65	2.08 ± 0.53	2.48 ± 0.63

Note: SD, standard deviations; 15_WMT, 15-day working memory training group; AC2, active control group.

#### Combination analysis for experiments 1 and 2

**Figure 5:**
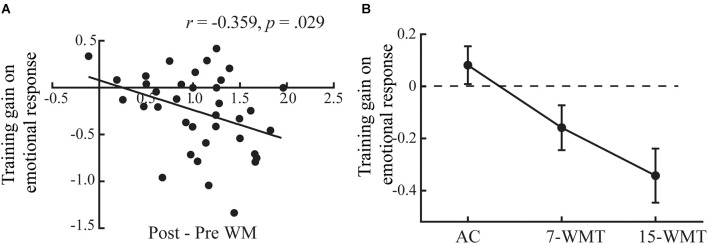
The results of combination analysis for Experiments 1 and 2. The correlation between training gains on the emotional response (training gain = posttest − pretest emotional response) and changes in WM in the training group **(A)**. The training gains on emotional response are plotted for three groups **(B)**. Error bars indicate standard errors. 7-WMT and 15-WMT represent the 7-day and 15-day working memory training groups, respectively.

To better investigate the training-induced changes in emotional response, we conducted a combination analysis using the data from Experiment 1 and Experiment 2. There was a significant linear correlation between pretest to posttest training gains for WM and reduction in emotional response (Figure 5A; *r* = −0.359, *p* = 0.029). This suggested that more significant gains from the WM training were associated with a higher reduction in negative emotional responses.

We further compared the training gain (posttest − pretest) of emotional response (Attend − Neutral) among the 7-WMT, 15-WMT, and active control groups (Figure 5B; *F*_(2,70)_ = 6.63, *p* = 0.002, $\eta_{p}^{2}$ = 0.159). *Post hoc* analyses revealed that the difference between the 7-WMT and AC groups was marginally significant (*p* = 0.06); the difference between the 15-WMT and AC groups was significant (*p* = 0.002). Although the difference between the 7-WMT and 15-WMT groups was nonsignificant (*p* = 0.195), reductions in emotional response were larger in the 15-WMT group (−0.343) than in the 7-WMT group (−0.159).

Finally, we conducted a three-way ANOVA using the emotional response data from the Anticipation Only trial to examine whether the WM training enhanced participants’ proactive emotion regulation. We found a significant Test Session (pretest, posttest) × Group (WMT, AC) × Cue (Neutral, Attend) interaction (*F*_(1,71)_ = 6.47, *p* = 0.013, $\eta_{p}^{2}$ = 0.084). WM training significantly reduced expected emotional response (Attend − Neutral) from the pretest to the posttest (*F*_(1,36)_ = 9.116, *p* = 0.005). However, no significant change was found in the AC group (*F*_(1,35)_ = 0.388, *p* = 0.538).

### General discussion

The present study investigated the transfer effect of non-emotional WM training on the emotional response by employing a training protocol. The Preliminary experiment in Supplementary Material demonstrated that the expectations about potential gains from the training tasks of the experimental group were matched to those of the active control group. The WM training successfully improved the adaptive response to negative stimuli in the following training. Specifically, the reduction of emotional response was marginally significant in the 7-day WM training group (Experiment 1), and significant in the 15-day WM training group (Experiment 2). In contrast, the active control group showed no changes in either 7-day or 15-day training procedures. Further, the combination analysis for Experiments 1 and 2 showed that the reduced emotional response significantly correlated with increased WM performance. Thus, the transfer effect displayed a dosage effect, i.e., increasing the amount of training was associated with a more profound improvement in emotional responses.

Cognitive training is a promising approach for cognitive enhancement; however, it remains controversial due to unelaborated experimental designs that may not preclude the influence of expectations. Considering the potential impact of expectations on the observed training effects (Foroughi et al., 2016; Long et al., 2020), it is necessary to match individuals’ expectations for intervention and control tasks (Boot et al., 2013; Ziegler et al., 2019). The Preliminary experiment was conducted to address this issue, and the results demonstrated that both tasks generated comparable training-related expectations of improvement in emotional control ability. Moreover, to preclude word-induced expectations during the training procedure, the participants did not receive suggestive phrases, such as “cognitive enhancement” or “brain training” (Foroughi et al., 2016). Therefore, the improvements observed in the current study could be exclusively attributed to WM training.

The present study suggested that non-emotional WM training could improve emotional response after matching the expectation about potential gains from the training tasks. Similarly, previous studies indicated that participants could decrease emotions after non-emotional cognitive training, i.e., inhibitory control training and attentional bias modification training (Beauchamp et al., 2016; Cohen et al., 2016; Hoorelbeke et al., 2016; Li et al., 2016; Peckham and Johnson, 2018). Working memory is a limited-capacity system involving a central executive and two storage systems (the phonological loop; the visuospatial sketchpad; Baddeley, 2003). The neural substrate of the central executive construed by the prefrontal cortex (PFC), basal ganglia, and thalamic, which are susceptible to be improved by working memory training (D’Esposito and Postle, 2015; Miro-Padilla et al., 2019; Finc et al., 2020). As working memory performance improved, individuals learned more adaptive ways of allocating resources. Besides, it has been found that individuals with high WM capacity tend to use cue information to prepare a response in advance (Redick, 2014; Wiemers and Redick, 2018). According to the DMC framework (Braver, 2012), proactive control is associated with sustained and anticipatory maintenance of relevant information within the PFC. Individuals can use proactive control to conduct anticipatory preparation for an upcoming cognitive task. Thus, the improved WM capacity may lead the participants to employ antecedent-focused emotional control strategies (e.g., situation modification, attentional deployment) to decrease negative emotion before it is fully processed (Gross, 2013; Martins-Klein et al., 2020).

Further, training on the *n*-back task could robustly activate the PFC (Owen et al., 2005; Constantinidis and Klingberg, 2016), which is also engaged during emotional control. The adaptive response depends on a balance between top-down cognitive control processes and bottom-up emotional responses (Ochsner et al., 2012). Results from a article we are preparing show that the connection between the emotion regulation network and amygdala is more robust in the high WM group than in the low WM group when they perform an emotional perception task. Thus, the transfer of WM training to emotional response may be due to the enhanced efficiency of the brain network.

The above interpretation is strengthened by the results of the Anticipation Only trials, which did not include emotional stimuli. Participants could feel negative emotions after they detected the cue because the cue of each trial could trigger emotional history. The results suggested that participants with WM training felt less emotional response (Attend − Neutral) at the posttest relative to the pretest. By contrast, no significant change was found in the active control group. Together, these results showed that the training group’s proactive response was indeed enhanced. The anticipatory preparation enabled participants to decrease negative emotions in advance, even when emotional stimuli were absent.

Notably, there was a dosage effect related to the influence of WM training on emotional response. We observed a marginal reduction in reported emotion following the 7-day WM training but a significant reduction after the 15-day training. The further combination correlation analysis confirmed that gains in emotional response significantly correlated with performance gains in WM training. Gathercole et al. (2019) proposed that the transfer was a consequence of acquiring complex cognitive skills that could be used to accomplish untrained tasks with similar demands. In the current study, individuals’ WM performance was better in the post-training than in the pre-training. They may engage in proactive control (Kane and Engle, 2002; Redick, 2014; Wiemers and Redick, 2018). The transfer occurs when proactive control is applied to an ER task. The execution of proactive control would increase automatically with the increase in WM performance. In this case, more extended training increases WM performance and a greater likelihood that participants can transfer skills to decrease reported emotion.

Although a prior study showed that emotional WM training improved the effectiveness of emotion regulation strategy (Schweizer et al., 2013), the current study did not find a significant transfer effect on emotion regulation. One possible reason is that the emotional materials we used at the pretest and posttest were static pictures, eliciting a low arousal response relative to video (Courtney et al., 2010). In the present study, non-emotional WM training decreased emotional reports in Attend and Regulate conditions. At the same time, decreasing negative emotions to zero is challenging, and even participants used the reappraisal strategy to regulate their emotions.

The current study implies that cognitive training has significant clinical implications. It is well known that individuals with emotional disorders (e.g., depression, anxiety) have a solid emotional susceptibility to negative information (Disner et al., 2011). Training without emotional information is vital for their physical and mental health. Besides, the neural mechanism of cognitive training needs to be clarified. Further studies are needed to explore the neural or physiological indices of cognitive training. It is also necessary to test the sex difference on these training effects using cognitive neuroscience methods because sex difference is an important factor in emotional processing. Finally, the ER task involving cue-task association may generate uncontrollable effects on emotion evaluation (Denny et al., 2014). The cue itself may trigger an emotional response that can interact with the subsequent stimulus; thus, this task may induce a cost for switching actions as the cues occur randomly. In the future, further studies could explore the transfer of WM training to mental health in a real-world setting to avoid this problem. For instance, using the experience sampling methodology to assess the affective states or psychopathological symptoms in daily life may elucidate transfer effects (Hoorelbeke et al., 2016; Boemo et al., 2022).

## Conclusion

Compared with the expectation-matched active control group, participants trained with the non-emotional dual *n*-back task could decrease their emotional response to negative stimuli, suggesting that individuals can anticipate and prepare for emotional events. Additionally, the results of different training durations (7-day and 15-day) indicated that WM training has a dosage effect on emotional response. Increased WM was significantly related to reduced emotional response, implying that the more training the participants received, the fewer negative emotions they would experience. Together, the current findings suggest that WM training may lead participants to execute proactive control when they face undesired situations, which has significant implications for clinical application.

## Data Availability Statement

The raw data supporting the conclusions of this article will be made available by the authors, without undue reservation.

## Ethics Statement

The studies involving human participants were reviewed and approved by The Human Research Ethics Application Form, Faculty of Psychology, Southwest University. The patients/participants provided their written informed consent to participate in this study.

## Author Contributions

QLo: conceptualization, methodology, and writing. LY, YT, and QLi: data curation and visualization. NH and YG: data analysis. AC: supervision, review, and editing.

## Footnotes

^1^ http://brainworkshop.net/
