# Supplementary material for: Improving adaptive response to negative stimuli through non-emotional working memory training
Source: Front Behav Neurosci. 2023 Jan 5;16:1058866. doi: 10.3389/fnbeh.2022.1058866 (PMC9851398; doi:10.3389/fnbeh.2022.1058866)
Supplement: Supplementary file 1 [file Presentation_1.pdf]

Supplementary Information

Preliminary experiment Expectation-Matched Placebo Control

This preliminary experiment aimed to assess whether the control task matched the working memory (WM) training task in terms of participants' expectations regarding potential gains of emotional control (Boot et al., 2013; Long et al., 2020).

Methods

Participants

A total of 33 healthy college students (21 women, aged 20 ± 0.25) were recruited to participate in Preliminary experiment. All participants signed written informed consent forms when they arrived at the lab and received monetary rewards after they completed all experiments. All procedures were performed following relevant guidance and were approved by the local ethical committee of Southwest University. The study was conducted in accordance with the Code of Ethics of the World Medical Association (Declaration of Helsinki) for experiments involving humans.

Stimuli and design

First, participants completed one block of the WM task (dual *n*-back) or the placebo task (visual search task). The detailed procedures of two tasks were presented in the manuscript (see *Stimuli and design* in Experiment 1). Participants then rated the

extent of improvements in emotional control using a self-report questionnaire. Next, they completed another task and its corresponding questionnaire. The sequence of the two tasks was counterbalanced across participants.

(1) The instruction for the dual *n*-back task

The following task is a dual *n*-back task. The following experiment shows a sequence of squares at eight different locations on the computer screen. Along with the presence of squares, a series of words will appear on the headset. If the current position matches the position presented *n* items back, you should press "A" on the keyboard; If the current word matches the word presented *n* items back, you should press "L". After the experiment, you need to answer four questions.

(2) The instruction for the visual search task

The following task is a search task. In this task, a series of stimuli will be presented on the computer screen, and you need to determine the orientation of the line segment in the diamond among circles. If the orientation is horizontal, you should press "F" on the keyboard; If the orientation is vertical, you should press "J" on the keyboard. After the experiment, you should finish a questionnaire.

(3) The questionnaire comprised of the following four questions:

a. Do you believe that emotional control ability is malleable? (*yes or no*)

b. How likely will your emotional control ability improve through the 7-day training on this task? (9-point scale; 1 = *not at all likely*, 5 = *neutral*, 9 = *extremely*

likely)

c. What do you think your score of emotional control ability is now? (100-point scale)

d. What will your score be if you practice this task for seven days? (100-point scale)

Data Analysis

A paired t-test was used to analyze the difference in the possibility of improvement between the WM and placebo tasks. The expected gain effect was represented by the difference between the current score of emotional control ability and the predicted score after training. We used a paired t-test to examine the difference in gain effect between the two tasks.

Results

All participants believed that both the WM (5.73) and placebo tasks (6.48) caused a slight improvement after a 7-day training procedure. The difference between the two tasks was not significant (Figure 1A; t (32) = 1.90, p = .067, *Cohen's d* = 0.672). The expected gain effect did not differ between the two tasks (Figure 1B; t (32) = 0.31, p = .761, *Cohen's d* = 0.110). These findings suggest that the placebo task matched the expectations of improvement with the WM task and therefore was an appropriate control condition. In Experiment 1 and Experiment 2, we adopted this placebo task (visual search task) as the training task of the active control group.

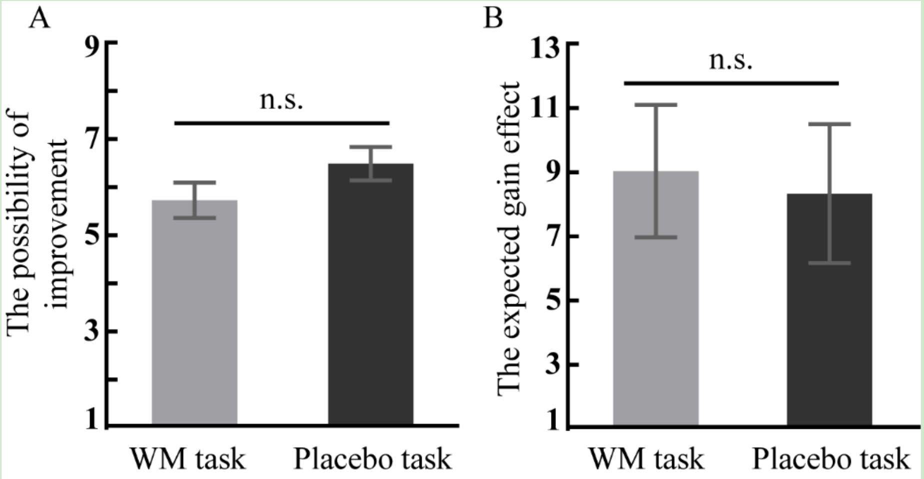

Figure 1. The expectations for WM task and placebo task. The possibility of improvement (A) and the expected gain effect (B; future score - now score) in Experiment 1. n.s. = non-significant.

References

Boot, W. R., Simons, D. J., Stothart, C., & Stutts, C. (2013). The Pervasive Problem With Placebos in Psychology: Why Active Control Groups Are Not Sufficient to Rule Out Placebo Effects. *Perspectives on Psychological Science A Journal of the Association for Psychological Science*, *8*(4), 445-454. https://doi.org/10.1177/1745691613491271

Long, Q., Hu, N., Li, H., Zhang, Y., Yuan, J., & Chen, A. (2020). Suggestion of cognitive enhancement improves emotion regulation. *Emotion*, *20*(5), 866-873. https://doi.org/10.1037/emo0000629
